# Integrated Phenotypic and Genomic Characterization of Cefotaxime/Clavulanic Acid Inhibitor-Positive Multidrug-Resistant *Escherichia coli* from Large-Scale Pig Farms in Hungary

**DOI:** 10.3390/ani16050722

**Published:** 2026-02-25

**Authors:** Ádám Kerek, Balázs Nagyházi, Gergely Álmos Tornyos, Levente Hunor Husz, Eszter Kaszab, Enikő Fehér, Patrik Mag, Ákos Jerzsele

**Affiliations:** 1 Department of Pharmacology and Toxicology, University of Veterinary Medicine, István utca 2, H-1078 Budapest, Hungary; nagyhazi.balazs@student.univet.hu (B.N.); tornyos.gergely.almos@student.univet.hu (G.Á.T.); husz.levente.hunor@student.univet.hu (L.H.H.); mag.patrik@univet.hu (P.M.); jerzsele.akos@univet.hu (Á.J.); 2 National Laboratory of Infectious Animal Diseases, Antimicrobial Resistance, Veterinary Public Health and Food Chain Safety, University of Veterinary Medicine, István utca 2, H-1078 Budapest, Hungary; kaszab.eszter@univet.hu (E.K.); feher.eniko@univet.hu (E.F.); 3 One Health Institute, University of Debrecen, Nagyerdei Krt. 98, H-4032 Debrecen, Hungary; 4 Department of Microbiology and Infectious Diseases, University of Veterinary Medicine, István utca 2, H-1078 Budapest, Hungary; 5 Department of Bioinformatics, University of Debrecen, Nagyerdei Krt. 98, H-4032 Debrecen, Hungary

**Keywords:** *E. coli*, extended-spectrum β-lactamase (ESBL), multidrug resistance, swine, whole-genome sequencing

## Abstract

**Background:** Extended-spectrum β-lactamase (ESBL)-producing *E. coli* are a major One Health concern because they compromise critically important cephalosporins and may spread via mobile genetic elements, including plasmids and transposon-associated modules, within food-animal production systems. **Objectives:** The aim of this study was to characterize cefotaxime (CTX)/clavulanic acid (CLA) inhibitor-positive phenotype profiles in pig-associated multidrug-resistant (MDR) *E. coli* and resolve their genetic basis using whole-genome sequencing, with emphasis on ESBL determinants and their predicted mobility context. **Methods:** MDR *E. coli* isolates (*n* = 203) from four large-scale pig farms in Hungary were tested by broth microdilution minimum inhibitory concentration (MIC) determination and Clinical and Laboratory Standards Institute (CLSI) inhibitor-based ESBL confirmation using cefotaxime with/without clavulanic acid. CTX/CLA inhibitor-positive isolates (inhibitor-positive phenotype) were subjected to whole-genome sequencing (WGS; *n* = 116) and resistome profiling; antimicrobial resistance genes (ARGs) were called against the Comprehensive Antibiotic Resistance Database (CARD) and mobility context was inferred using plasmid-origin and MGE-proximity prediction. **Results:** Overall, 127/203 isolates (62.6%) showed a CTX/CLA inhibitor-positive phenotype with a pronounced inhibitory effect (median cefotaxime/cefotaxime–clavulanate ratio: 33.3). In the sequenced subset (*n* = 116), 5427 ARG hits were identified (82 unique ARGs in the export), including frequent acquired determinants affecting tetracyclines, sulfonamides/trimethoprim, aminoglycosides, and phenicols; plasmid-mediated quinolone resistance (*qnrB5*) and mobilizable colistin resistance (*mcr-1*) were detected at low frequency. Classical β-lactamase genes were detected, including *CTX-M* (ESBL genes) and *TEM* alleles. *CTX-M* and/or *TEM* were detected in 47/116 genomes (40.5%), dominated by *CTX-M-32* (11.2%) and *TEM-1* (23.3%); detected ESBL determinants were predominantly predicted to be located on contigs predicted to be of plasmid origin, with a subset showing signatures consistent with transposition-associated mobilization. **Conclusion:** The high burden of inhibitor-positive phenotype, together with an inferred plasmid-/MGE-associated context for a subset of ESBL genes, and substantial phenotype–genotype heterogeneity, supports integrated phenotypic–genomic surveillance to refine AMR risk assessment and guide targeted stewardship and biosecurity interventions in pig production.

## Simple Summary

Antimicrobial resistance is a growing One Health concern because resistant bacteria and their resistance genes can circulate between animals, humans, and the environment. In pig production, *E. coli* is a key indicator organism and an important reservoir for resistance traits, including extended-spectrum β-lactamases (ESBLs) that reduce the effectiveness of critically important cephalosporins. Here, we investigated multidrug-resistant *E. coli* from large-scale pig farms in Hungary using standardized phenotypic testing and whole-genome sequencing to clarify the genetic basis of cefotaxime (CTX)/clavulanic acid (CLA) inhibitor-positive phenotypes. Among 203 isolates, 62.6% showed a CTX/CLA inhibitor-positive phenotype with a strong clavulanate inhibitory effect. Whole-genome sequencing of 116 CTX/CLA inhibitor-positive isolates detected classical ESBL-associated β-lactamase gene families (*CTX-M* and/or *TEM*) in 40.5% of genomes, and these determinants were predominantly predicted to be located on contigs of putative plasmid origin, suggesting a potentially mobile genetic context, although horizontal transfer was not demonstrated in this study. Notably, many isolates with a CTX/CLA inhibitor-positive phenotype lacked these classical ESBL gene calls, highlighting that inhibitor-based phenotypes can reflect heterogeneous and complex resistance architectures. Our findings underscore the value of combining phenotypic methods with genome-resolved interpretation to strengthen AMR surveillance and to support targeted antimicrobial stewardship and biosecurity interventions in pig production.

## 1. Introduction

Antimicrobial resistance (AMR) is widely recognized as a major and escalating threat to global health, food security, and sustainable animal production, driven by the combined selective pressure of antimicrobial use in human and veterinary medicine and the capacity of bacteria to acquire and disseminate resistance determinants [1,2,3]. Within food-producing systems, antimicrobials have historically been used not only for therapy but also, in some settings, for metaphylaxis and prophylaxis, creating sustained selection that favors multidrug-resistant (MDR) bacterial populations [4,5]. Because resistant bacteria and resistance genes circulate at the human–animal–environment interface, AMR is best addressed through a “One Health” framework that integrates surveillance and interventions across sectors [6,7,8,9].

Beyond public-health relevance, AMR also imposes measurable economic costs on livestock production through increased morbidity and mortality, reduced productivity, higher prevention and treatment expenditures, and potential disruption of trade in animal products. Macroeconomic modelling suggests that, without effective containment, AMR could reduce annual global GDP by 1.1% in a low-impact scenario and up to 3.8% in a high-impact scenario by 2050; output and trade in livestock and livestock products are projected to be particularly vulnerable, with low-income settings facing the largest relative losses. These projections further support the economic rationale for integrated AMR containment and surveillance investments across human and animal sectors [10].

Among livestock sectors, pig production represents a substantial consumer of antimicrobials globally, reflecting intensive production systems, high animal densities, and the need to control endemic bacterial disease [11,12,13]. National usage patterns can markedly deviate from European averages, underscoring the importance of country-specific surveillance and stewardship strategies [14]. Harmonized monitoring approaches for zoonotic and indicator bacteria have therefore become a cornerstone of European AMR risk assessment, enabling comparisons across time, regions, and production types and supporting targeted mitigation efforts [15,16]. In this context, robust data from large-scale pig farms are critical, as they inform veterinary decision-making [7,15].

*E. coli* is a central organism in AMR epidemiology because it is ubiquitous in the intestinal microbiota of animals and humans; it can act as a primary pathogen in multiple syndromes, and it serves as an efficient reservoir and vehicle for horizontal gene transfer of antimicrobial resistance genes (ARGs) [17,18,19]. The dissemination of ARGs can extend beyond farms through fecal shedding and environmental contamination, contributing to cyclic “farm-to-environment-to-farm” dynamics and increasing the likelihood that resistance determinants reach the food chain and, ultimately, human populations [2,3,20,21,22]. Importantly, the transfer of resistance plasmids across hosts has long been documented, illustrating that the movement of mobile genetic elements can link animal and human bacterial communities [23,24,25].

A particularly consequential AMR phenotype in *E. coli* is the production of extended-spectrum β-lactamases (ESBLs), enzymes that confer resistance to extended-spectrum cephalosporins and compromise treatment options in both veterinary and human medicine [26,27]. ESBL determinants—most notably within the *CTX-M* family, alongside *TEM*-derived variants—have diversified and expanded worldwide, with food-producing animals and their environments increasingly recognized as relevant reservoirs [28,29]. Within the European Union, ESBL-producing *E. coli* have been repeatedly reported in pigs and pork, and the farm-level ecology of ESBL carriage appears sensitive to antimicrobial use patterns and stewardship interventions [30,31,32,33]. Pig production is also relevant because occupational exposure has been associated with ESBL carriage in farm workers and pig farmers, supporting the plausibility of bidirectional transmission routes at the farm interface [34,35].

Accurate detection and characterization of ESBL-producing *E. coli* are therefore essential for surveillance and for guiding prudent antimicrobial use [7,15]. However, inhibitor-positive phenotype testing can be challenging in the presence of alternative β-lactam resistance mechanisms, particularly plasmid-mediated AmpC enzymes or chromosomal *ampC* overexpression, which may obscure or mimic ESBL phenotypes and reduce the reliability of routine confirmatory tests [36,37]. In addition, the clinical implications of ESBL and/or AmpC production can vary across strain backgrounds and resistance combinations, emphasizing the need for approaches that resolve underlying mechanisms rather than relying solely on phenotype [28,38]. In European pig production, ESBL-/AmpC-producing *E. coli* are repeatedly detected, and their reported prevalence varies widely across countries and study designs. For example, EU/EEA monitoring data for fattening pigs have shown country-level proportions ranging from 1.0% to 85.3% (2020–2021), highlighting substantial heterogeneity in their epidemiological background and surveillance approaches. Hungarian data also indicate a considerable ESBL burden in porcine samples, supporting the relevance of farm-level One Health-oriented surveillance in this setting [39,40].

From a swine management and treatment perspective, ESBL-associated resistance compromises the clinical utility of extended-spectrum cephalosporins (e.g., ceftiofur/cefquinome), which are considered critically important antimicrobials and are recommended for restricted, susceptibility-guided use in veterinary medicine. Reduced effectiveness of these agents can constrain empiric therapy for *E. coli*–associated disease (including enteric syndromes) and may increase reliance on alternative antimicrobial classes, reinforcing the need for prevention-oriented interventions (e.g., vaccination where applicable, hygiene, all-in/all-out flow, segregation, cleaning/disinfection, and movement control) alongside diagnostics and antimicrobial stewardship [41,42,43,44,45].

Whole-genome sequencing (WGS) and related next-generation sequencing (NGS) approaches offer a high-resolution framework to address these challenges by enabling the comprehensive identification of resistance determinants, the characterization of plasmid- and mobile-element-associated genes, and the contextual interpretation of complex phenotypic results [46,47]. Beyond mechanistic attribution, genome-based AMR profiling has suggested the potential to predict antimicrobial susceptibility from sequence data and to strengthen surveillance pipelines by linking genotype, phenotype, and epidemiological context [15,48,49]. In swine production specifically, WGS-based investigations have proven valuable for simultaneous assessment of ESBL-associated genes and broader AMR/virulence repertoires across animal and environmental compartments of farms [50].

Against this backdrop, the present study aimed to characterize MDR *E. coli* isolated from large-scale pig farms in Hungary, with a specific focus on ESBL production, by integrating phenotypic testing with WGS-based genomic analysis [15,50]. Beyond reporting prevalence, we explicitly quantify phenotype–genotype heterogeneity under a standardized (CTX)/clavulanic acid (CLA) inhibitor-based definition and interpret resistome findings under conservative, short-read-aware assumptions. This provides a practical surveillance framework that separates inhibitor-positive phenotypes from gene-confirmed ESBL determinants and highlights where gene-centric calling is likely to under-explain phenotype. The resulting dataset offers regionally relevant, age-stratified insight from large-scale pig production in Hungary. We sought to quantify and describe the inhibitor-positive phenotype in a large isolate collection, identify ESBL-encoding gene families and related β-lactam resistance determinants through WGS, and explicitly address phenotypic–genotypic discrepancies by considering alternative β-lactamase mechanisms that can confound ESBL detection. By combining standardized phenotyping with genome-resolved interpretation, our work provides a mechanistic baseline for surveillance and supports evidence-based antimicrobial stewardship in pig production.

## 2. Materials and Methods

### 2.1. Study Design, Farm Origin, and Isolate Collection

Ethical review and approval were waived for this study because no animals were sampled specifically for research purposes and no additional procedures were performed; all isolates were retrieved retrospectively from routine veterinary diagnostic submissions and subsequent institutional strain banking.

This study investigated MDR *E. coli* isolates originating from commercial, high-throughput (“large-scale”) pig production systems in Hungary, with a primary focus on the CTX/CLA inhibitor-positive phenotype and its genomic background. According to the strain-bank records, isolates were originally obtained by attending farm veterinarians as part of routine diagnostic activities and were submitted for bacteriological work-up within standard veterinary workflows. Isolates were collected over an approximately four-week period in late 2023 (November 2023). The originating holdings were located in three Hungarian regions (Nyugat-Dunántúl, Dél-Dunántúl, and Dél-Alföld); however, detailed farm-level metadata (e.g., herd size, antimicrobial usage, and biosecurity indicators) were not available for this retrospective diagnostic collection. Sampling was convenience-based within routine diagnostic submissions rather than a pre-planned systematic survey. Importantly, each isolate originated from a different individual pig (one isolate per animal); no repeated isolates from the same animal were included. Clinical status at the time of submission was not captured in a fully standardized manner across all submissions, whereas farm of origin and production stage/age category were recorded.

Specimens were transported to the Department of Epidemiology and Microbiology, University of Veterinary Medicine Budapest, using charcoal-containing Amies transport swab systems (Biolab Zrt., Budapest, Hungary). Following bacteriological isolation, pure cultures were provided for further analyses. Isolates were cryopreserved at −80 °C using the Microbank bead-based system (Pro-Lab Diagnostics, Richmond Hill, Canada) until testing. A total of 203 *E. coli* isolates were included in the phenotypic workflow. Here, the phenotypic workflow comprised standardized broth microdilution MIC testing across the selected antimicrobial panel. ESBL phenotype was then assessed by CTX/CLA inhibitor-based confirmation and interpreted using Clinical and Laboratory Standards Institute (CLSI) criteria. Species confirmation was subsequently achieved using matrix-assisted laser desorption ionization–time of flight (MALDI-TOF) mass spectrometry (Flextra-LAB Kft., Budapest, Hungary) and the Biotyper software, version 12.0 (Bruker Daltonics GmbH, Bremen, Germany; 2024 release).

### 2.2. Minimum Inhibitory Concentration (MIC) Determination

Minimum inhibitory concentrations (MICs) were determined prior to isolate selection for whole-genome sequencing, following the CLSI recommendations. CLSI breakpoints were used for resistance categorization where applicable [51].

Briefly, isolates stored at −80 °C were inoculated into 3 mL cation-adjusted Mueller–Hinton broth (CAMHB; Biolab Zrt., Budapest, Hungary) and incubated at 37 °C for 18–24 h. MIC testing was performed in sterile 96-well microtiter plates (VWR International, LLC., Debrecen, Hungary), prefilled with 90 µL CAMHB per well. Antibiotic stock solutions (Merck KGaA, Darmstadt, Germany) were prepared at 1024 µg/mL in accordance with CLSI guidance [52]. For each agent, 90 µL of the stock solution was dispensed into the first column, followed by two-fold serial dilutions across the plate. After the 10th column, excess volume was removed, resulting in a final volume of 90 µL per well.

Bacterial suspensions were adjusted to 0.5 McFarland using a nephelometer (Thermo Fisher Scientific, Budapest, Hungary). A 10 µL inoculum was added to each well as described in the CLSI-aligned workflow. Plates were incubated at 37 °C for 18–24 h, and MIC endpoints were read using the Sensititre SWIN automated reading system together with the VIZION system, version 3.4 (Thermo Fisher Scientific, Budapest, Hungary). Quality control was performed using *E. coli* ATCC 25922.

β-Lactamase activity was screened using a nitrocefin-based chromogenic cephalosporin assay (Cefinase-type). Nitrocefin is hydrolyzed by β-lactamases with a rapid yellow-to-red color change and is widely used as a sensitive, generic activity screen for β-lactamase production; however, it is not ESBL-specific, and its detectability may vary with enzyme type and expression level. In a recent Enterobacterales-focused evaluation of a nitrocefin-based biochemical assay, nitrocefin hydrolysis detected all ESBL producers (22/22) and all carbapenemase producers (36/36) at 10^5^ CFU/mL, while remaining negative in all non-β-lactamase producers (15/15), supporting high sensitivity for clinically important β-lactamases and high specificity for absence of β-lactam hydrolysis [53,54]. Fresh overnight growth was used to prepare a heavy inoculum, which was applied to nitrocefin-impregnated disks/strips and observed for a color change from yellow to red. A color change within 5 min at room temperature was interpreted as β-lactamase-positive, while no color change within 15 min was considered negative. Quality control included *E. coli* ATCC 25922 (negative control) and *E. coli* ATCC 35218 (positive control). Results were recorded as a binary variable (positive/negative) and summarized by farm and age group.

### 2.3. CTX/Clavulanate Inhibitor-Based Phenotype Definition

Inhibitor-positive phenotype confirmation was performed according to the CLSI-recommended procedure [52]. For clarity, “CTX/CLA inhibitor-positive phenotype” refers solely to an inhibitor response in the CLSI ESBL confirmatory test performed with cefotaxime ± clavulanic acid and does not imply genetic confirmation; ESBL status is reserved for isolates with WGS-detected ESBL genes. MICs were determined for cefotaxime alone and for the cefotaxime–clavulanic acid combination, maintaining a constant clavulanic acid concentration of 4 µg/mL across all dilutions in the combination wells. Plates were incubated at 37 °C for 18–24 h, and isolates were interpreted as ESBL producers when the cefotaxime–clavulanic acid MIC was reduced by ≥3 two-fold dilution steps (i.e., ≥8-fold) compared with cefotaxime alone, in accordance with CLSI criteria. Inhibitor-positive-phenotype-positive isolates were prioritized for whole-genome sequencing.

Ceftazidime ± clavulanate was not included in the applied confirmatory panel; therefore, the phenotype definition should be interpreted as CTX-based inhibitor positivity. 

### 2.4. Genomic DNA Extraction and Whole-Genome Sequencing

Genomic DNA was extracted using the Quick-DNA Fungal/Bacterial Miniprep Kit (Zymo Research, Irvine, CA, USA) according to the manufacturer’s instructions. Mechanical lysis was performed by bead beating using a TissueLyzer LT (Qiagen GmbH, Hilden, Germany) at 50 Hz for 5 min. Extracted DNA was stored at −20 °C until further processing.

Paired-end whole-genome sequencing was performed by Novogene (Cambridge, UK) on an Illumina NextSeq 500 platform, using Illumina sequencing-by-synthesis chemistry. Libraries were prepared using the Nextera XT DNA Library Preparation Kit (Illumina, San Diego, CA, USA), and dual indexing was performed with the Nextera XT Index Kit v2 Set A (Illumina, San Diego, CA, USA) i5/i7 primer pairs. Briefly, DNA was normalized to 0.2 ng/µL (2.5 µL input), and tagmentation was carried out at 55 °C for 6 min, followed by neutralization at room temperature for 5 min. Indexed libraries were amplified using 12 PCR cycles (95 °C for 10 s, 55 °C for 30 s, and 72 °C for 30 s), preceded by an initial denaturation step (95 °C for 30 s) and followed by a final extension step (72 °C for 5 min). Libraries were purified using the Geneaid Gel/PCR DNA Fragments Extraction Kit (Geneaid Biotech Ltd., New Taipei City, Taiwan), quantified with the Qubit dsDNA HS Assay Kit (Thermo Fisher Scientific, Waltham, MA, USA), pooled, and sequenced.

### 2.5. Read Processing, Genome Assembly, and Quality Assessment

Raw read quality was evaluated using FastQC v0.11.9, and reads were preprocessed using fastp v0.23.2-3 and Bloocoo v1.0.7 [55,56,57]. Adapter and quality trimming were performed using Trim Galore v0.6.6 [58]. De novo assemblies were generated using MEGAHIT v1.2.9 and SPAdes v4.0.0, and the resulting assemblies were merged using GAM-NGS v1.1b to obtain a robust draft genome per isolate [59,60,61].

Assembly quality and completeness were evaluated with QUAST v5.2 and BUSCO v5 [62,63]. Genome-level characteristics were estimated using GenomeScope v2.2 based on k-mer distributions [64].

Assembly quality metrics (contig count, N50, total length, GC%) are provided in Supplementary Table S1 to contextualize downstream plasmid-origin inference.

### 2.6. Resistome Calling and Mobility Inference

Open reading frames were predicted using Prodigal v2.6.3 [65]. Antimicrobial resistance genes (ARGs) were identified using Resistance Gene Identifier (RGI) v5.1.0 and ABRicate against the Comprehensive Antibiotic Resistance Database (CARD) [66]. Only hits meeting the CARD STRICT criterion and showing ≥90% sequence identity and ≥90% coverage were retained [47]. These conservative thresholds were selected to prioritize high-confidence ARG annotation for surveillance-grade interpretation. For per-isolate prevalence analyses, multiple hits corresponding to the same ARG in a given genome were collapsed to a single presence call to avoid double counting; fragmented/partial matches below thresholds were excluded.

Associations between ARGs and mobile genetic elements (MGEs) were assessed using MobileElementFinder v1.0.3; ARGs were classified as mobile when located within the organism-specific maximum transposon distance defined in the underlying database [67]. Plasmid origin of contigs was inferred using PlasFlow v1.1 [68]. For both MGE- and plasmid-associated signals, only predictions supported by contigs of at least 10,000 bp were considered. Species confirmation was performed using CheckM v1.2.2 and Kraken v1.1.1 [69,70].

Because mobility context was inferred from short-read assemblies, plasmid architecture and horizontal transfer potential cannot be confirmed without long-read sequencing and/or conjugation experiments. A schematic overview of the study design and analytical workflow is provided in Figure S1.

### 2.7. In Silico MLST

In silico multilocus sequence typing (MLST) was performed from assembled genomes using the *E. coli* Achtman 7-locus scheme. Alleles and sequence types (STs) were assigned based on full-length matches; MLST results were summarized at the ST level to describe lineage diversity within the sequenced subset.

## 3. Results

### 3.1. Isolate Set and Phenotypic Screening Outcomes

In total, 203 *E. coli* isolates were recovered from four large-scale pig farms in Hungary: Farm 1 in Dél-Dunántúl (*n* = 58), Farm 2 in Nyugat-Dunántúl (*n* = 70) and Farm 3 and Farm 4 in Dél-Alföld (*n* = 75). The cohort comprised isolates originating (Table 1) from three age groups (day-old piglets, *n* = 72; 4-week-old piglets, *n* = 65; and 6-week-old piglets, *n* = 66). CTX/CLA inhibitor-based confirmatory testing classified 127/203 isolates (62.6%) as having an inhibitor-positive phenotype (CTX-based), while β-lactamase positivity was recorded for 110/203 isolates (54.2%). Inhibitor-positive phenotype frequency varied across farms, ranging from 38.5% to 86.1%, and was highest among day-old piglets (75.0%) compared with 4-week-old (52.3%) and 6-week-old piglets (59.1%).

### 3.2. Phenotypic MIC Distributions Across the Antimicrobial Panel

**Figure 1:**
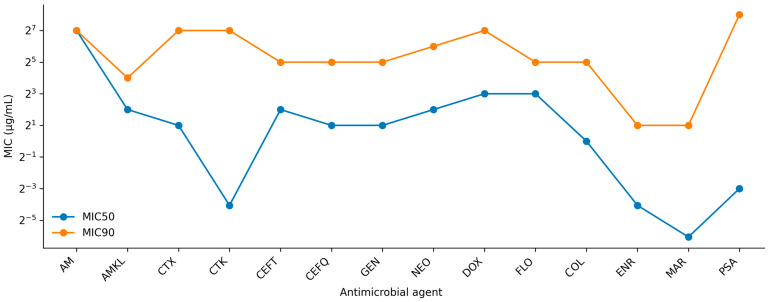
MIC_50_ and MIC_90_ across the antimicrobial panel (*n* = 203). MIC_50_ and MIC_90_ represent the 50th and 90th percentiles of the minimum inhibitory concentration (MIC) distributions for each antimicrobial. Values are shown on a log2-scaled y-axis (µg/mL). AM—amoxicillin, AMKL—amoxicillin–clavulanic acid, CTX—cefotaxime, CTK—cefotaxime–clavulanic acid, CEFT—ceftiofur, CEFQ—cefquinome, GEN—gentamicin, NEO—neomycin, DOX—doxycycline, FLO—florfenicol, COL—colistin, ENR—enrofloxacin, MAR—marbofloxacin, PSA—potentiated sulfonamide (trimethoprim–sulfamethoxazole).

Broth microdilution revealed wide MIC distributions across the tested antimicrobial classes. At the cohort level, amoxicillin showed a high central tendency (MIC_50_/MIC_90_: 128/128), whereas fluoroquinolone MICs were lower on average (enrofloxacin MIC_50_/MIC_90_: 0.06/2; marbofloxacin MIC_50_/MIC_90_: 0.015/2). For third- and fourth-generation cephalosporins, cefotaxime and ceftiofur exhibited elevated upper-tail MICs (cefotaxime MIC_50_/MIC_90_: 2/128; ceftiofur MIC_50_/MIC_90_: 4/32). Colistin MICs displayed a broad range (MIC_50_/MIC_90_: 1/32), and trimethoprim–sulfamethoxazole showed pronounced heterogeneity (MIC_50_/MIC_90_: 0.125/256). Full MIC distributions are provided in Supplementary Table S2. Figure 1 shows the MIC_50_ and MIC_90_ across the antimicrobial panel. Table 2 summarizes the antimicrobial panel.

### 3.3. CTX/CLA Inhibitor-Based Testing Demonstrated a Robust Clavulanate Effect in CTX/CLA Inhibitor-Positive Isolates

**Figure 2:**
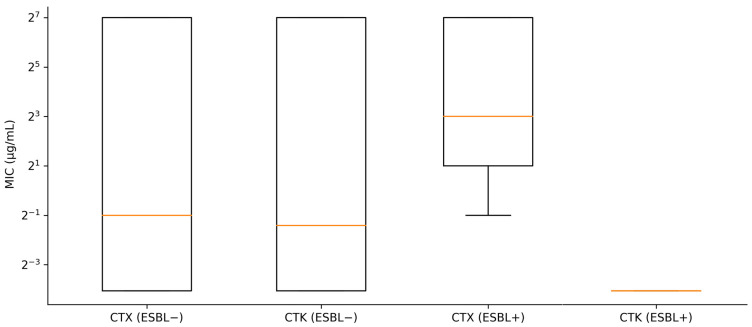
Cefotaxime (CTX) and cefotaxime–clavulanic acid (CTK) minimum inhibitory concentration (MIC) distributions stratified by CTX/CLA inhibitor-based phenotype (*n* = 203). Boxplots show median and interquartile range; whiskers represent 1.5 × IQR. MICs are shown on a log2-scaled y-axis (µg/mL). −: negative; +: positive.

A pronounced clavulanate-associated MIC reduction was observed in CTX/CLA inhibitor-positive isolates (Figure 2). In the CTX/CLA inhibitor-positive group, the cefotaxime MIC_50_ was 8, while the cefotaxime–clavulanic acid MIC_50_ was 0.06, consistent with inhibitor-based phenotypic confirmation. In contrast, the CTX/CLA inhibitor-negative group showed a median cefotaxime/cefotaxime–clavulanic acid ratio of 1.0, whereas the inhibitor-positive group exhibited a substantially higher median ratio (33.3), indicating a strong inhibitory effect in the confirmed ESBL phenotype.

Cross-classification of the CTX/CLA inhibitor-positive phenotype status and nitrocefin β-lactamase screening showed that 76/127 (59.8%) ESBL phenotype-positive isolates were β-lactamase-positive, whereas 51/127 (40.2%) were β-lactamase-negative. Among ESBL phenotype-negative isolates, 34/76 (44.7%) were β-lactamase-positive.

### 3.4. WGS Resistome Overview in the Sequenced Subset

Whole-genome sequencing and resistome calling were performed for 116 CTX/CLA inhibitor-positive isolates (inhibitor-positive phenotype). Across this sequenced subset, 5427 ARG hits were identified, corresponding to an average of 46.8 hits per isolate and spanning 82 unique resistance-associated genes in the provided export. Several resistance-related loci were ubiquitous and largely consistent with a conserved chromosomal “core” background (e.g., multidrug efflux and regulatory determinants). In addition, multiple acquired-resistance determinants were detected at variable frequencies, including tetracycline (*tet* variants), sulfonamide (*sul* variants), trimethoprim (*dfr* variants), aminoglycoside-modifying enzymes, phenicol determinants (*floR* and *catI*), plasmid-mediated quinolone resistance (*qnrB5*), and the mobilizable colistin resistance determinant *mcr-1*.

### 3.5. Acquired-Resistance Determinants Beyond β-Lactams in the Sequenced Subset

**Figure 3:**
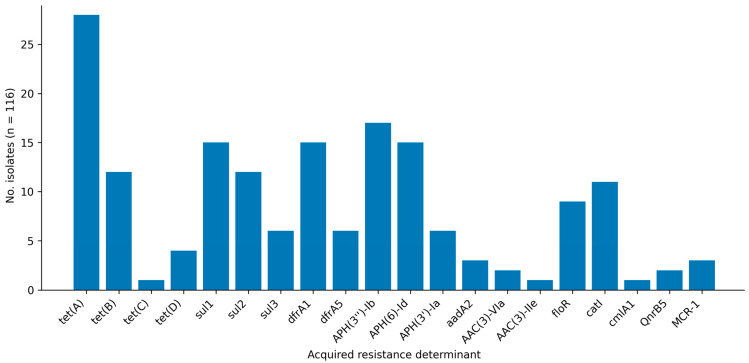
Prevalence of selected acquired-resistance genes in the sequenced CTX/CLA inhibitor-positive subset (*n* = 116). Bars indicate the number of isolates carrying each gene (presence/absence). Only selected acquired determinants of direct interpretive relevance to the tested panel and high-priority traits are shown.

Across the 116 sequenced isolates, acquired-resistance determinants were detected for multiple antimicrobial classes (Table 3 and Figure 3). Tetracycline resistance genes were frequent, with *tet(A)* identified in 28/116 isolates (24.1%), *tet(B)* in 12/116 (10.3%), *tet(D)* in 4/116 (3.4%) and *tet(C)* in 1/116 (0.9%). Sulfonamide resistance determinants were also common (*sul1*, 15/116; 12.9%; *sul2*, 12/116; 10.3%; *sul3*, 6/116; 5.2%), and trimethoprim resistance genes were detected at comparable frequencies (*dfrA1*, 15/116; 12.9%; *dfrA5*, 6/116; 5.2%). Aminoglycoside-modifying enzymes occurred in a subset of isolates, including *APH(3*″*)-Ib* (17/116; 14.7%), *APH(6)-Id* (15/116; 12.9%), and additional aminoglycoside-associated genes at lower prevalence. Phenicol-associated genes were detected, including *floR* (9/116; 7.8%) and *catI* (11/116; 9.5%). Plasmid-mediated quinolone resistance was infrequent (*qnrB5*, 2/116; 1.7%). Notably, the mobilizable colistin resistance determinant *mcr-1* was present in 3/116 isolates (2.6%).

### 3.6. ESBL-Associated β-Lactamase Determinants and Their Predicted Mobility Context

**Figure 4:**
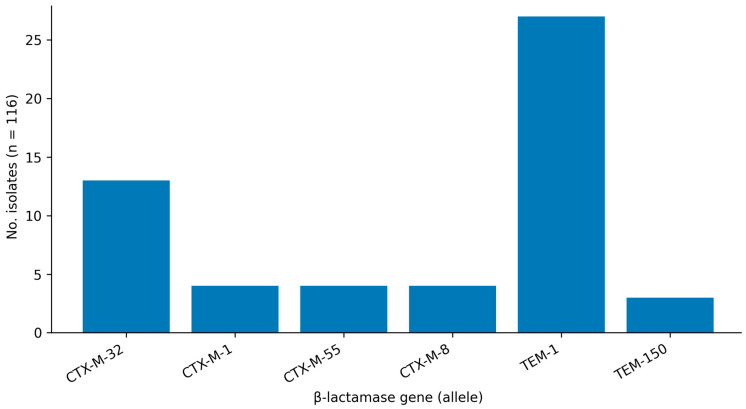
Distribution of extended-spectrum β-lactamase (ESBL)-associated β-lactamase alleles in the sequenced subset (*n* = 116). Bars indicate the number of isolates carrying each allele (presence/absence). Mobility context was inferred from plasmid-origin prediction and mobile genetic element (MGE)-proximity signals derived from short-read assemblies.

Within the sequenced subset, *CTX-M*-type β-lactamase genes were detected in 24/116 isolates (20.7%). The most frequent *CTX-M* allele was *CTX-M-32* (13/116; 11.2%), while *CTX-M-1, CTX-M-55*, and *CTX-M-8* each occurred in 4/116 isolates (3.4% each). *TEM*-type β-lactamase genes were identified in 30/116 isolates (25.9%), dominated by *TEM-1* (27/116; 23.3%), with *TEM-150* detected in 3/116 isolates (2.6%). Co-occurrence of *CTX-M* and *TEM* genes was observed in seven isolates (Figure 4).

PlasFlow-based classification indicated that contigs carrying *CTX-M* and *TEM* determinants were predominantly predicted to be of plasmid-origin. Mobile genetic element (MGE) proximity analysis further suggested that a subset of these determinants resided within 10 kb of insertion sequences or transposons. Specifically, *CTX-M-32* was located within 10 kb of ISKpn26 in 2/13 isolates, *CTX-M-8* was linked to an IS26-associated composite transposon in 1/4 isolates, *TEM-1* was located within 10 kb of Tn2 or IS26 in 7/27 isolates, and *TEM-150* showed proximity to Tn801 in 2/3 isolates. Collectively, these analyses suggest that CTX-M and TEM determinants detected in this study are predominantly located on contigs predicted to be of plasmid origin, and that a subset shows proximity signatures consistent with transposition-associated contexts (Table 4). These findings represent inferred mobility context from short-read assemblies and do not demonstrate plasmid architecture or horizontal transferability.

### 3.7. Genomic Resolution of ESBL Phenotypes and Mechanistic Heterogeneity

**Figure 5:**
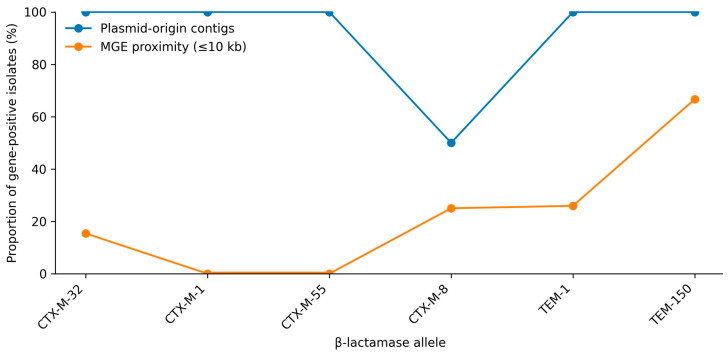
Predicted mobility context of extended-spectrum β-lactamase (ESBL)-associated β-lactamase determinants (*n* = 116). Plasmid origin indicates the proportion of isolates in which a given determinant was detected on a contig classified as plasmid-derived; mobile genetic element (MGE) proximity indicates the proportion with a mobile element identified within ≤10 kb.

Although all 116 sequenced isolates originated from the phenotypic CTX/CLA inhibitor-positive phenotype, *CTX-M* and/or *TEM* determinants were identified in 47/116 isolates (40.5%) in the ARG output, leaving 69/116 isolates (59.5%) without detectable *CTX-M* or *TEM* alleles under the applied calling criteria (Figure 5). In parallel, chromosomal *ampC*-associated loci were near-ubiquitous (*E. coli ampC* detected in 115/116 isolates; *E. coli ampC1* β-lactamase detected in 106/116 isolates), alongside a conserved background of multidrug efflux and regulatory determinants in the resistome output. These findings indicate that, within this inhibitor-positive phenotype group cohort, WGS-based ARG calling identified “classical” ESBL gene families in a substantial minority of isolates, while the majority lacked detectable *CTX-M/TEM* alleles, underscoring the importance of genome-resolved interpretation when inhibitor-positive phenotype confirmation is used as a selection gate for downstream characterization.

### 3.8. Population Structure by MLST

MLST of the 116 sequenced isolates (Supplementary Table S3) revealed high lineage diversity, with 54 distinct sequence types (STs) identified. No single ST dominated the dataset; the most frequent lineages were ST58 (11/116; 9.5%), ST137 (9/116; 7.8%), ST10 (8/116; 6.9%), and ST641 (6/116; 5.2%), while additional STs occurred at lower frequencies. This distribution is consistent with a heterogeneous population structure in the sequenced subset, suggesting that the inhibitor-positive phenotype is not restricted to a single clonal background.

## 4. Discussion

In this study, we provide a phenotype-to-genotype resolved view of third-generation cephalosporin non-susceptibility in *E. coli* from large-scale Hungarian pig production, using a large phenotypic panel (*n* = 203) coupled to targeted WGS of phenotypically CTX/CLA inhibitor-positive isolates (*n* = 116). The most striking finding is the high proportion of inhibitor-based ESBL phenotypes (62.6%), which while broadly consistent with reports describing 30–50% ESBL-producing *E. coli* in pig farms and pork-associated isolates in parts of Europe, still places these production settings at the high end of what is typically reported, underscoring the need for sustained, farm-level AMR control and surveillance [30,31].

A second key message is that the genomic “confirmation layer” fundamentally sharpens interpretation: among the 116 phenotypic inhibitor-positive phenotype group isolates with adequate DNA for sequencing, classical ESBL gene families were detected in 40.5% (47/116).

This magnitude aligns closely with an extensive European investigation reporting that 45% of ESBL-producing isolates from pigs carried ESBL genes, suggesting that phenotypic selection gates can substantially enrich isolates with ESBL-like behavior while still capturing a sizable fraction whose third-generation cephalosporin phenotype is driven by alternative mechanisms and/or remains unresolved with gene-centric calling alone [35].

From a One Health perspective, this matters because policy and risk communication often rely on simplified phenotypic labels; our data show that “ESBL phenotype” can represent a composite of mechanisms with different mobility and transmission potential, which should be explicitly recognized in surveillance pipelines and intervention strategies [6,7]. The farm- and age-stratified patterns add biological plausibility and practical relevance. Inhibitor-positive phenotype frequency was highest in day-old piglets and declined in older age groups, consistent with early-life colonization and rapid acquisition of resistant Enterobacterales via the sow, the environment, and management-associated contact [32,33].

This early-life burden is especially important because neonatal colonization can set the trajectory of the gut resistome and creates an early window where biosecurity, hygiene, and antimicrobial stewardship could have disproportionately large effects [5,11,12]. At the same time, β-lactamase activity varied markedly by farm and age group, indicating heterogeneous β-lactam resistance backgrounds across production settings and suggesting that selection and persistence of β-lactam resistance determinants are not restricted to the earliest production stage, reinforcing the need for longitudinal on-farm monitoring rather than single-timepoint screening.

At the gene-family level, our ESBL landscape is notable for the predominance of *CTX-M* and *TEM* determinants, but with a distribution that differs from the “canonical European” picture. While *CTX-M-1* is widely described as a dominant lineage in multiple European contexts, we observed *CTX-M-32* and *CTX-M-55* among the most frequent alleles in our sequenced subset, with *TEM-1* also common [28,71,72,73]. Such heterogeneity is in line with the broader literature showing substantial between-country and between-study variation, likely reflecting differences in antimicrobial use practices, farm management, animal movement networks, and methodological approaches (including sampling frame and ESBL confirmation strategy) [34,73,74,75]. Importantly, this is not merely a descriptive nuance: different ESBL alleles can be embedded in distinct plasmid backbones and mobilization contexts, which directly influences transfer rates, persistence under fluctuating selection, and spillover into humans [17,24,25].

Indeed, one of the most policy-relevant aspects of our dataset is the predicted mobility context of ESBL determinants. *CTX-M-1* was detected exclusively on plasmid-associated contigs; *CTX-M-32* and *CTX-M-8* appeared frequently in plasmid contexts but also showed MGE linkage in a subset, and *TEM* family members displayed substantial MGE association (e.g., *TEM-1* was frequently linked to MGE) [67,68,71]. This configuration is consistent with large-scale farms functioning as environments where plasmid- and IS/transposon-associated resistance modules may persist and circulate within the production ecosystem. However, in our dataset these conclusions remain inferential, as plasmid backbones and transfer were not resolved or experimentally assessed; therefore, any dissemination beyond the farms should be interpreted as a plausible risk pathway rather than confirmed transmission [34,35]. The implication of these results is forward-looking: risk mitigation should prioritize not only reducing selection pressure (e.g., stewardship and prudent use) but also interrupting the ecological and contact networks that facilitate MGE exchange, particularly in early life stages where colonization is established [5,11,12,17,24,25].

The predominance of *CTX-M-32* observed here is consistent with reports from European piggeries where *CTX-M-1* group variants, including *CTX-M-32*, circulate across diverse *E. coli* lineages but are frequently linked to successful conjugative plasmid backbones (notably IncI1/ST3 and IncN/ST1), supporting a predominantly plasmid-driven dissemination model in swine production settings [76]. In parallel, *TEM-1* is a widespread narrow-spectrum β-lactamase in animal-associated *E. coli* and other Enterobacterales, often embedded in transposon- and IS26-rich resistance regions (e.g., Tn2/Tn3-family contexts) that facilitate reshuffling and co-selection on MDR plasmids; *TEM-1*-containing IS26/Tn2 modules have also been documented in swine-farm-associated Enterobacterales beyond *E. coli* (e.g., *Salmonella*) [77].

Within swine farm systems, dissemination of these determinants can plausibly occur via both clonal expansion of successful lineages and horizontal transfer of plasmids or transposon modules within the gut microbiota, and in high-density microbial matrices such as manure and slurry. Environmental persistence and recirculation routes—contaminated pen surfaces, equipment, water lines, personnel movement, and manure storage/land application with subsequent soil/runoff contamination—can further amplify cross-compartment spread and create opportunities for re-introduction into the herd [78]. Given that our mobility signals are inferred from short-read proximity and plasmid-origin prediction, these pathways should be interpreted as biologically plausible routes supported by prior literature rather than directly demonstrated within the present dataset [79].

The discrepancy between a robust CTX/CLA inhibitor-positive phenotype and the absence of *CTX-M/TEM* calls in a substantial fraction of sequenced isolates warrants cautious interpretation. Several non-mutually exclusive explanations may contribute to this pattern, including chromosomal mechanisms affecting β-lactam susceptibility (e.g., *ampC* regulatory variation), altered outer-membrane permeability (e.g., porin loss-of-function), and efflux/regulatory changes, as well as technical constraints inherent to gene-centric ARG calling and short-read assemblies. Importantly, these chromosomal promoter/attenuator variants, regulatory alterations, membrane permeability changes, and expression-level effects were not experimentally assessed in this study; therefore, they are discussed here strictly as hypotheses and were not inferred from the current dataset. In addition, plasmid-mediated AmpC families were not detected under the applied ARG-calling criteria, but we note that AmpC-mediated phenotypes remain a general consideration in inhibitor-based screening workflows. First, gene-centric AMR calling has known limitations; detection depends on assembly continuity, database representation of alleles, and parameter thresholds, and it does not capture regulatory variation, gene expression, or chromosomal mutation-driven resistance [47,66]. Second, β-lactam resistance in *E. coli* is multi-layered and can involve chromosomal cephalosporinase activity (e.g., *ampC* axis), porin changes, and efflux upregulation, which can shift MIC distributions and interact with inhibitor-based tests in ways that complicate categorical interpretation [27,36,37,38,73,80,81,82,83,84]. In our cohort, the widespread presence of chromosomal *ampC*-related loci in the resistome output supports the plausibility that non-classical or composite mechanisms contributed to the third-generation cephalosporin phenotype in at least a subset of isolates [37,38]. Importantly, plasmid-mediated AmpC families (e.g., *CMY*, *DHA*, *FOX*, *MOX*, *ACC*) were not detected under our applied CARD/RGI filtering, arguing against pAmpC as a dominant explanation in this sequenced subset. However, promoter or attenuator variants affecting chromosomal *ampC* expression, porin loss-of-function (*ompC, ompF*), and regulatory changes influencing efflux are not captured by gene-centric ARG calling; therefore, these mechanisms remain plausible but unconfirmed contributors to the observed *CTX* and *CLA* inhibitor-positive phenotype.

One plausible contributor to this apparent ESBL phenotype–genotype discordance is AmpC-mediated resistance, particularly chromosomal *ampC* promoter/attenuator mutations leading to *ampC* overexpression, which can elevate cefotaxime MICs without introducing a “new” acquired ARG detectable by gene-centric pipelines [85]. In addition, plasmid-mediated AmpC (e.g., CMY-type) can produce extended-spectrum cephalosporin non-susceptibility and complicate inhibitor-positive phenotype workflows; however, no plasmid-mediated AmpC families were detected under our applied calling/filters, so this mechanism cannot be confirmed in our dataset [86]. Finally, inhibitor-based confirmation tests are known to have methodological limitations and may yield discordant or occasionally false-positive ESBL interpretations depending on the underlying β-lactamase background and co-occurring permeability/efflux changes; accordingly, we interpret inhibitor-positivity as a phenotypic signal that may reflect heterogeneous resistance architectures rather than gene-confirmed ESBL production in every isolate [36].

Methodologically, these findings argue for an integrated interpretive workflow: inhibitor-based phenotyping remains valuable for initial triage, but WGS interpretation should ideally extend beyond acquired gene detection to include chromosomal mutational mechanisms and context-aware inference (e.g., porin/efflux signatures and regulatory variants) if the goal is to explain phenotype at isolate resolution [47,48,66].

Beyond β-lactams, the sequenced isolates carried a broad set of acquired-resistance determinants (e.g., tetracyclines, sulfonamides, trimethoprim, aminoglycoside-modifying enzymes, and phenicols), consistent with the concept that selection for ESBL plasmids often co-selects multi-class resistance through physical linkage and shared mobility platforms [17,24,25]. The detection of *mcr-1* in a small subset is particularly important even at low frequency, because mobilizable colistin resistance is a recognized high-concern trait, and its co-occurrence potential with ESBL determinants has been documented in pig-associated *E. coli* [75,87]. Meanwhile, the pervasive identification of multidrug efflux and regulatory determinants highlights that the apparent “baseline” resistome architecture of production-associated *E. coli* can provide a permissive background for acquired genes to express clinically meaningful phenotypes under selection [88].

When positioned against European surveillance benchmarks, our results sit at an instructive intersection: inhibitor-positive phenotype positivity was high, while the WGS-confirmed presence of classical ESBL genes among phenotypically positive isolates (40.5%) is broadly comparable to European-scale observations and to reports across diverse settings (including Asia and Latin America), where prevalence varies widely and is frequently explained by differences in antimicrobial usage and study design [71,72,75]. For Hungary, this supports a dual conclusion. First, large-scale farms can harbor a substantial burden of cephalosporin-related resistance phenotypes and mobile ESBL gene pools, suggesting a plausible pathway for dissemination, while acknowledging that the present study infers mobility from short-read data and does not confirm transfer [34,35,71]. Second, risk assessment and intervention design should not equate “ESBL phenotype” with “classical ESBL genotype” without genomic verification, because the underlying mechanisms and transferability may differ materially [36,37,38,47,66,67].

Putting our findings into a regional context, ESBL/extended-spectrum cephalosporin resistance in pig-associated *E. coli* has been repeatedly documented across Central Europe. A longitudinal production-cycle investigation from the Czech Republic recovered CTX-M-producing *E. coli* across multiple age categories and demonstrated conjugative plasmids carrying *CTX-M* variants, supporting persistence and dissemination potential within pig production systems. These observations align with our inference that at least a subset of ESBL determinants in our dataset is associated with mobile contexts, while simultaneously underscoring that the inhibitor-positive phenotype signal can be detected across diverse genomic backgrounds and may not be fully captured by “classical ESBL gene” calls alone.

Several limitations also delineate the next steps that could elevate this work from a robust national snapshot to a mechanistic, high-impact One Health contribution. The WGS subset was derived from phenotypic CTX/CLA inhibitor-positive phenotype isolates, so genomic prevalence estimates are conditioned on that selection gate and should not be extrapolated to the full 203-isolate cohort without appropriate weighting. In addition, short-read assemblies constrain plasmid reconstruction and can fragment MGE contexts; long-read sequencing of representative *CTX-M-32/CTX-M-55/TEM-150* carriers would enable complete plasmid resolution, replicon typing, and direct confirmation of transposon structures and insertion sequence contexts that are only inferred here [17,24,25,67,68]. Accordingly, the plasmid location, backbone structure, and true horizontal transferability of these determinants remain inferred rather than demonstrated; definitive confirmation would require long-read plasmid reconstruction and/or conjugation experiments. While MLST indicates substantial lineage diversity, higher-resolution phylogeny (cgMLST/SNP) integrated with plasmid epidemiology would further clarify whether observed ESBL determinant patterns reflect clonal expansion, plasmid epidemicity, or repeated introductions [46,48]. In addition, we did not evaluate *ampC* promoter/attenuator mutations, porin gene disruptions, or efflux regulatory variants through targeted variant analysis or functional assays; thus, chromosomal mechanisms remain unresolved in this dataset.

In Enterobacterales, *CTX-M* genes commonly occur in transposition units linked to insertion sequences that can facilitate capture and movement onto plasmids, and reports describe *CTX-M-32* within IS-associated contexts consistent with transposition-mediated dissemination. Likewise, *TEM-1* is frequently embedded in Tn3-family transposons (e.g., Tn2), and IS26 is widely implicated in restructuring resistance regions via composite-transposon-like arrangements and recombination. Importantly, in our dataset these interpretations are based on short-read proximity signatures rather than resolved element boundaries [89,90].

Farm-level antimicrobial usage data and detailed biosecurity indicators were not available for this strain-bank-based dataset, limiting causal attribution of between-farm and between-age-group differences. Nonetheless, the high frequency of CTX/CLA inhibitor-positive phenotypes supports reinforcing antimicrobial stewardship and core biosecurity measures that reduce fecal–oral transmission within and between production stages (e.g., hygiene barriers, age-group segregation/all-in–all-out management, manure handling practices, and personnel/equipment separation). This CTX-based inhibitor approach may not fully discriminate all AmpC- or inhibitor-resistant mechanisms, which may contribute to phenotype–genotype discordance in a subset of isolates [91].

From a management perspective, our results support translating surveillance outputs into targeted stewardship and biosecurity reinforcement [92]. Farm-level longitudinal data indicate that the occurrence of ESBL-producing *E. coli* is shaped not only by antimicrobial selection pressure but also by specific management practices, implying that coordinated reductions in the use of critically important cephalosporins; strict all-in/all-out flow; effective cleaning and disinfection between batches; segregation of sick animals; and tight control of movements of personnel, vehicles and equipment can reduce transmission and persistence. Modeling work similarly highlights that preventing introduction onto farms and ensuring rapid isolation of affected animals are high-impact levers [33,93]. Although detailed farm-level antimicrobial usage and biosecurity metadata were unavailable for our retrospective diagnostic collection, incorporating these parameters into future integrated surveillance would substantially improve risk attribution and the design of intervention bundles.

Overall, our findings suggest that large-scale pig production can act as a reservoir where inhibitor-positive cephalosporin resistance phenotypes are common and where a subset of CTX-M/TEM genes shows inferred plasmid-/MGE-associated context. The extent of horizontal transferability and dissemination cannot be confirmed without long-read reconstruction and/or conjugation assays, and we therefore frame dissemination as a risk consideration rather than a demonstrated outcome [71]. The early-life enrichment of ESBL phenotypes points to a critical intervention window, and the observed phenotype–genotype complexity argues for surveillance frameworks that combine standardized inhibitor-based testing with genome-resolved interpretation of both acquired genes and chromosomal mechanisms [36,37,38,66,67]. Such an integrated approach is the most direct path to translating AMR measurements into actionable control strategies that reduce both animal health burdens and the broader public health risk associated with antimicrobial resistance dissemination through the food chain and occupational exposure [6,7,34,44,74,94].

## 5. Conclusions

This study provides a phenotype-to-genotype assessment of third-generation cephalosporin resistance in pig-associated multidrug-resistant *E. coli* from large-scale farms in Hungary. CTX/CLA inhibitor-positive phenotypes were frequent in the overall cohort (127/203; 62.6%) and showed a strong inhibitory effect, indicating a substantial burden of ESBL-like β-lactam resistance in these production settings. Whole-genome sequencing of the CTX/CLA inhibitor-positive subset (*n* = 116) identified *CTX-M* and/or *TEM* β-lactamase determinants in 47/116 isolates (40.5%) and indicated that detected ESBL-associated genes were predominantly located on contigs predicted to be of plasmid origin, with additional signatures consistent with MGE-mediated mobilization in a subset. Beyond β-lactams, the presence of multiple acquired-resistance determinants (e.g., tetracycline, sulfonamide/trimethoprim, aminoglycoside-modifying enzymes, and phenicols) supports the likelihood of co-selection and persistence under on-farm antimicrobial exposure.

Importantly, the discordance between inhibitor-based ESBL phenotypes and the absence of “classical” ESBL gene families in the majority of sequenced isolates underscores that inhibitor-positive phenotype confirmation can reflect heterogeneous genetic architectures, including non-classical or composite resistance mechanisms that are not fully captured by gene-centric calling alone. Collectively, these findings argue for integrated surveillance workflows that pair standardized inhibitor-based phenotyping with genome-resolved interpretation, including mobility context, to more accurately quantify transmission risk and to guide targeted stewardship and biosecurity interventions, particularly in early production stages where inhibitor-positive phenotype frequency was highest. Future work leveraging long-read sequencing and combining chromosomal/plasmid epidemiology will be critical to fully resolving plasmid backbones, transposon structures, and dissemination routes and thereby translating these observations into actionable One Health risk-reduction strategies.

## Figures and Tables

**Table 1:** Cohort structure and screening outcomes by farm and age group. β-lactamase positivity was determined by a nitrocefin-based chromogenic cephalosporin test. ESBL—extended-spectrum β-lactamase. CTX/CLA inhibitor-positive phenotype was defined by cefotaxime with/without clavulanate (CLSI inhibitor-based confirmation).

Farm	Age Group	*n*	CTX/CLA Inhibitor-Positive, *n* (%)	β-Lactamase-Positive, *n* (%)
Farm 1	1-day	23	14 (60.9)	20 (87.0)
Farm 1	4-week	17	3 (17.6)	12 (70.6)
Farm 1	6-week	18	9 (50.0)	10 (55.6)
Farm 2	1-day	22	19 (86.4)	15 (68.2)
Farm 2	4-week	24	20 (83.3)	20 (83.3)
Farm 2	6-week	24	16 (66.7)	22 (91.7)
Farm 3	1-day	15	12 (80.0)	1 (6.7)
Farm 3	4-week	12	0 (0.0)	0 (0.0)
Farm 3	6-week	12	3 (25.0)	1 (8.3)
Farm 4	1-day	12	9 (75.0)	0 (0.0)
Farm 4	4-week	12	11 (91.7)	0 (0.0)
Farm 4	6-week	12	11 (91.7)	9 (75.0)
Farm 1	Total	58	26 (44.8)	42 (72.4)
Farm 2	Total	70	55 (78.6)	57 (81.4)
Farm 3	Total	39	15 (38.5)	2 (5.1)
Farm 4	Total	36	31 (86.1)	9 (25.0)
All farms	1-day	72	54 (75.0)	36 (50.0)
All farms	4-week	65	34 (52.3)	32 (49.2)
All farms	6-week	66	39 (59.1)	42 (63.6)
All farms	Overall	203	127 (62.6)	110 (54.2)

**Table 2:** Antimicrobial panel and minimum inhibitory concentration (MIC) summary statistics (*n* = 203).

Antimicrobial	Class	MIC Range (µg/mL)	MIC_50_	MIC_90_
Amoxicillin	Penicillins	1–128	128	128
Amoxicillin–clavulanic acid	Penicillins/β-lactamase inhibitors	1–32	4	16
Cefotaxime	3rd-generation cephalosporins	0.06–128	2	128
Cefotaxime–clavulanic acid	3rd-generation cephalosporins/β-lactamase inhibitors	0.06–128	0.06	128
Ceftiofur	3rd-generation cephalosporins	0.06–32	4	32
Cefquinome	4th-generation cephalosporins	0.015–32	2	32
Gentamicin	Aminoglycosides	0.5–128	2	32
Neomycin	Aminoglycosides	1–128	4	64
Doxycycline	Tetracyclines	0.5–128	8	128
Florfenicol	Phenicols	2–128	8	32
Colistin	Polymyxins	0.5–32	1	32
Enrofloxacin	Fluoroquinolones	0.015–32	0.06	2
Marbofloxacin	Fluoroquinolones	0.015–32	0.015	2
Trimethoprim–sulfamethoxazole	Folate pathway inhibitors	0.125–256	0.125	256

**Table 3:** Prevalence of selected acquired-antimicrobial-resistance genes in the sequenced CTX/CLA inhibitor-positive subset (*n* = 116).

Gene	Antimicrobial Class	*n* (%)
*ANT(3*″*)-IIa*	Aminoglycosides	23 (19.8)
*APH(3*″*)-Ib*	Aminoglycosides	17 (14.7)
*APH(6)-Id*	Aminoglycosides	15 (12.9)
*APH(3*′*)-Ia*	Aminoglycosides	6 (5.2)
*ANT(2*″*)-Ia*	Aminoglycosides	6 (5.2)
*aadA2*	Aminoglycosides	3 (2.6)
*AAC(3)*-VIa	Aminoglycosides	2 (1.7)
*AAC(3)-IIe*	Aminoglycosides	1 (0.9)
*qacH*	Biocides (QAC)	3 (2.6)
*qnrB5*	Fluoroquinolones	2 (1.7)
*fosA7*	Fosfomycin	1 (0.9)
*catI*	Phenicols	11 (9.5)
*floR*	Phenicols	9 (7.8)
*cmlA1*	Phenicols	1 (0.9)
*mcr-1*	Polymyxins (colistin)	3 (2.6)
*sul1*	Sulfonamides	15 (12.9)
*sul2*	Sulfonamides	12 (10.3)
*sul3*	Sulfonamides	6 (5.2)
*tet(A)*	Tetracyclines	28 (24.1)
*tet(B)*	Tetracyclines	12 (10.3)
*tet(D)*	Tetracyclines	4 (3.4)
*tet(C)*	Tetracyclines	1 (0.9)
*dfrA1*	Trimethoprim	15 (12.9)
*dfrA5*	Trimethoprim	6 (5.2)

**Table 4:** Extended-spectrum β-lactamase (ESBL)-associated β-lactamase determinants and predicted mobility context in the sequenced subset (*n* = 116). MGE—mobile genetic element.

Gene	*n* (%)	Plasmid-Origin *n* (%)	Chromosome-Origin *n* (%)	MGEs Within 10 kb *n* (%)	Most Frequent MGEs (≤10 kb)	MGE Types (≤10 kb)
*CTX-M-1*	4 (3.4)	4 (100.0)	0 (0.0)	0 (0.0)	—	—
*CTX-M-32*	13 (11.2)	13 (100.0)	0 (0.0)	2 (15.4)	ISKpn26 (*n* = 2)	insertion sequence (*n* = 2)
*CTX-M-55*	4 (3.4)	4 (100.0)	0 (0.0)	0 (0.0)	—	—
*CTX-M-8*	4 (3.4)	2 (50.0)	2 (50.0)	1 (25.0)	cn_3765_IS26 (*n* = 1)	composite transposon (*n* = 1)
*TEM-1*	27 (23.3)	27 (100.0)	0 (0.0)	7 (25.9)	Tn2 (*n* = 6); IS26 (*n* = 1)	unit transposon (*n* = 6); insertion sequence (*n* = 1)
*TEM-150*	3 (2.6)	3 (100.0)	0 (0.0)	2 (66.7)	Tn801 (*n* = 2)	unit transposon (*n* = 2)

## Data Availability

The datasets used and/or analyzed during the current study are available from the corresponding author on reasonable request. The sequencing data are available in the NCBI BioProject database under accession number PRJNA1404818 (accessed on 8 February 2026).

## Supplementary Materials

The following supporting information can be downloaded at: https://www.mdpi.com/article/10.3390/ani16050722/s1. Table S1: Genome assembly quality metrics and ARG-calling post-processing rules. Per-isolate genome assembly statistics (e.g., number of contigs, total assembly length, GC content, N50/L50 and related fragmentation descriptors) for the sequenced dataset (*n* = 116), reported to contextualize plasmid-origin inference from short-read assemblies. Table S2: Full broth microdilution minimum inhibitory concentration (MIC) dataset for all *E. coli* isolates included in the study (*n* = 203), including isolate metadata (farm and age group) and MIC values for the complete antimicrobial panel. Table S3: Multilocus sequence typing (MLST) profiles of sequenced *E. coli* isolates. MLST results for all whole-genome sequenced isolates (*n* = 116). Sequence types (STs) were assigned in silico from assembled genomes using the Achtman *E. coli* MLST scheme. Figure S1: Overview of the study design and analytical workflow.
