# Supplementary material for: Integrated Phenotypic and Genomic Characterization of Cefotaxime/Clavulanic Acid Inhibitor-Positive Multidrug-Resistant Escherichia coli from Large-Scale Pig Farms in Hungary
Source: Animals (Basel). 2026 Feb 25;16(5):722. doi: 10.3390/ani16050722 (PMC12984990; doi:10.3390/ani16050722)
Supplement: Supplementary file 1 [file animals-16-00722-s001.zip › Supplementary Figure S1.pdf]

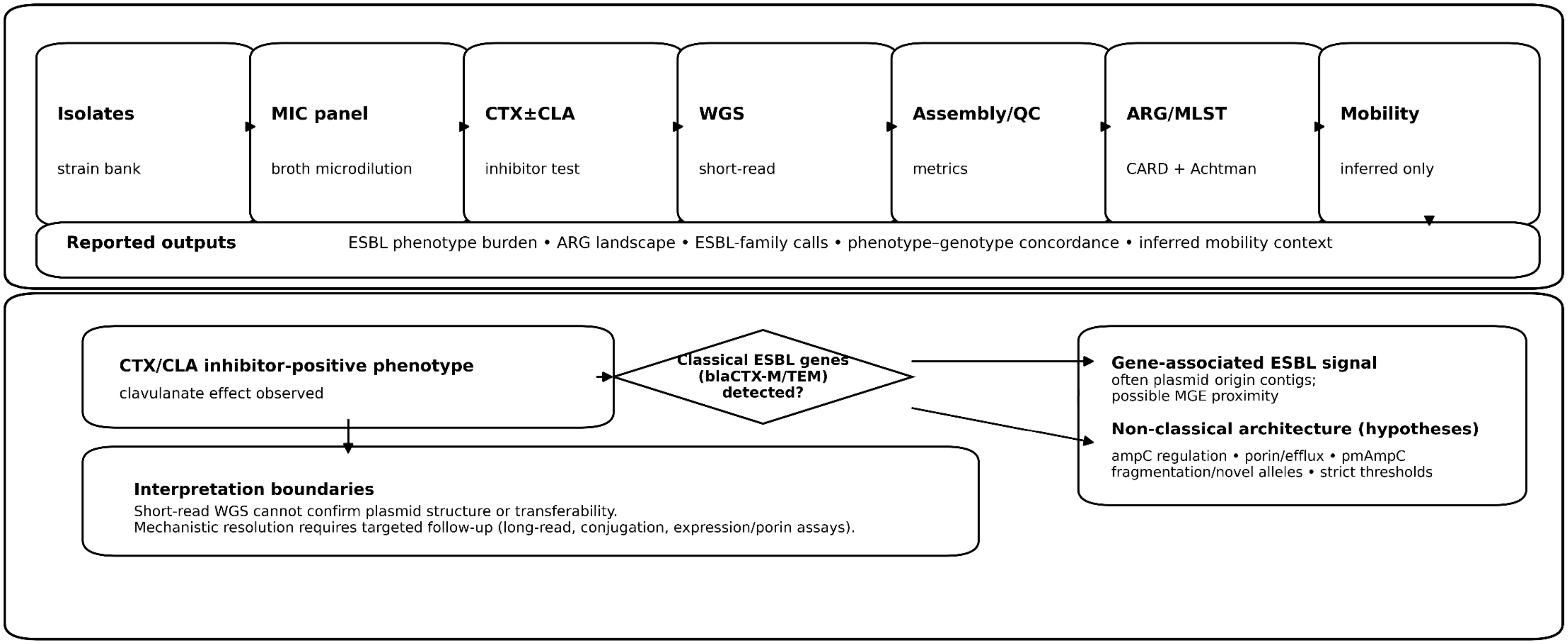

Supplementary Figure S1. Overview of the study design and analytical workflow. The schematic summarizes isolate origin (strain bank), phenotypic minimum inhibitory concentration (MIC) testing and CTX/CLA inhibitor-based phenotype definition, followed by whole-genome sequencing (WGS), assembly quality control, antimicrobial resistance gene (ARG) calling, and short-read-based mobility context inference used for the phenotype–genotype comparison
